# Progress in Medicine

**Published:** 1896-02-01

**Authors:** 


### PROGRESS IN MEDICINE.

### INFECTIVE DISEASES.

(*Continued from page* 251.)

**Tetanus.**—Sidney Martin[12] obtained from the blood and spleen of infected animals albumoses and certain organic acids. Some of the latter, which were soluble in ether, appear to be the specific poison, and produce muscular spasms and death when injected. Nocard, Roux, and Vaillard[13] have produced immunity, lasting for some weeks, by vaccinating with the serum of horses who had received gradually increased doses of the toxin. This has become of practical use in saving a large number of horses and sheep in certain localities where they are frequently attacked after slight wounds or castration, though it is useless when the disease has once appeared. Nestor Tirard[14] administered anti-toxin to a child suffering from severe tetanus three days after the spasms began. Five ten-grain doses in all were given, and complete recovery followed. Thirty-nine cases in which this remedy had been used were collected by F. H. Marson[15] in the autumn, and of these fourteen were reported as having died. Nine of them were classed as severe, and here the mortality was four out of the number.

**Rabies.**—Golgi[16] describes certain definite lesions occurring in the nuclei, nerve cells, neuroglia and ganglion cells. They all show commencing cell division, but these changes in the ganglion cells pass over into degeneration. The method of treatment by serum, as opposed to Pasteur's intensive vaccination, has been expounded by Tizzoni and Centanni.[17] The virus is first attenuated by peptic digestion, and then repeatedly injected into sheep. The serum thus formed will not only give immunity, but a small dose will neutralise the effect of previous infection, even if delayed till half the incubation period has passed. The virus acts chiefly on the central nervous system, and the serum exerts its force more powerfully if injected—at least in rabbits—under the dura mater than when used hypodermically. Between the two in efficacy is an injection made into a large nerve trunk. The serum is believed to strengthen the cells so that they can resist the effects of the poison, rather than to directly neutralise it. The authors advise that, after a human being has been bitten, 20 c.cm. of the serum, or a corresponding amount of the dried product, should be injected within four days. If it is delayed longer, then a double quantity is needed, and must be injected between the fourth and the fifteenth day. In any case half the dose is injected first and the remainder on two other days within a week. The paper gives a full account of the preparation and the long continued work done in determining the strength and action of the serum. In conclusion, we may add that, in spite of the difficulties of Pasteur's treatment[18]—which the serum method will, it is hoped, overcome—the mortality has been reduced by it from 15 to 0·92 per cent. in patients bitten by animals certified to be mad.

**Influenza.**—Lamarque[19] holds that the poison often acts on the kidneys, sometimes causing transient albuminuria, sometimes hæmorrhagic nephritis, and sometimes œdema and acute Bright's disease. Even death from uræmia may ensue. Cystitis and atony of the bladder, as well as uterine hæmorrhage, are among the other complications met with. Franke[20] reports twenty cases in which periostitis and inflammation of the plantar fasciæ and joints followed influenza, making the act of standing very painful. The insertion of the tendo Achillis is specially liable to attack. From the occasional abscesses in the lung found in this disease pure cultivations of the Pfeiffer's bacillus were obtained by Hitzig,[21] in place of the usual strepto- or staphylo coccus. J. Terry[22] describes a special appearance of the tongue as an almost universal mark of influenza. He finds it spotted over with purplish-red elevations, which later on change to vesicles, and these in turn often become white. Similar vesicles are to be found on the mucous membrane of the mouth. This weakened epithelium easily becomes the seat of various secondary infections.

**Typhoid.**—Balogness[23] praises the cold bath treatment, especially for patients in later life. He commences with baths at 65 degrees every three hours, and increases them to every two hours if symptoms of heart failure or hyperpyrexia arise. If necessary cold affusion to the spine or an icebag to the head are added. Jaquet[24] points out that in fevers there is a diminution of the red corpuscles in the circulation, which are meanwhile stored up in the liver and other organs, while cold baths overcome this tendency, so that after a bath we find an increase of those in the circulation even to the extent of 500,000 per cm. Further researches on the diazo reaction are recorded by Ettles.[25] He finds it invariably in typhoid and pneumonia (of course at certain stages only), less often in measles, acute intestinal cases, phthisis, and erysipelas. It does not as a rule occur in chronic or malignant non-tubercular visceral affections, but is not unknown even in perfect health. Serum injections have been used with very sensible relief in grave cases of typhus when employed in an early stage of the disease. The serum was obtained by Legrain[26] from two patients who had recovered from severe attacks. Beumer and Peiper[27] have injected sheep with typhoid cultures sterilised by moderate heat, and after several repetitions have obtained a serum which showed a curative action on animals. However, the course of the disease in them is so different to what occurs to man that actual experimental proof of its value is needful. The serum has, it is said, no bad effect on healthy men. Klemperer and Levy have utilised a similar serum for typhoid patients, and in these the disease ran a mild course after the injections. The feeding of typhoid and other fever patients on nothing but water for several days at the height of the attack is again recommended by A. M. Lesser[28] who reports good recoveries in eight cases which were fed with nothing but water for from five to fourteen days. He sometimes washes out the stomach at the beginning of the attack, and employs enemata of tepid water and mild antiseptics in addition. M'Cormick[29] contributes another paper on the treatment by saline purgatives which he sums up as follows: "Never allow the bowels to become constipated, never give opium, phenacetin, or acetanilide; give plenty of water by the rectum and the mouth, keep the alimentary canal as aseptic as possible, and give good nourishing food." The colon was washed out daily, and if the temperature was high ice-cold water was used, and guiacol applied locally. Iced-water injections were also used if hæmorrhage occurred. Quill[30] had only two deaths out of thirty-six cases in India, where a mortality of 20 to 30 per cent. is common. He employs three-minim doses of carbolic acid in compound tincture of chloroform every two or three hours. A. P. Hull praises the constant use of guiacol internally, and when needed externally, together with minute and repeated doses of calomel at first. F. H. Wiggin, in a paper he has recently published on perforation in enteric fever, finds that surgical interference has been attempted in seventeen cases of certain diagnosis, and that three only recovered; but he argues that with due precautions a much better result may be hoped for, and warmly recommends the operation. Search should be made in the pelvis, and the perforation closed by Lembert or Halstead's sutures; or even excision of a portion of the gut, and the use of Murphy's suture may be advisable. Creagh[31] reports a very rare complication—hæmoptysis, which occurred in a non-phthisical patient with much violence, and returned again and again till death took place. Epistaxis is comparatively frequent, and the writer considers that both depend on lessened coagulability of the blood. Alfred Parsons[32] describes another case of typhoid where acute œdema of the glottis took place, necessitating tracheotomy. Emphysema followed, and spread down the neck and trunk. The patient died after a few hours. No definite ulcers were found in the trachea. H. C. Parsons[33] describes six cases of post-typhoid bone necrosis, the lesions appearing in from one to sixteen months after convalescence. Three of the cases showed the lesions in the ribs, two in the tibiæ, and one in the left radius and right tibia. In four cases the typhoid bacillus was obtained in pure culture, and he remarks that the organisms have been thus found seven years after the fever. Even after the abscess is opened the bacilli may remain in it for a long period. Hence he advises complete excision of all diseased and suspected tissues. Yanowski[34] has recently found a case of suppurative parotitis in which the typhoid bacillus was present alone. Other previous cases have always shown other germs in addition to or without the typhoid one.

[12] B. M. J., Sept. 28. [13] Lancet, Nov. 2. [14] Lancet, Nov. 2. [15] Lancet, Aug. 10. [16] Am. M. S. Bulletin, Sept. 15. [17] Lancet, Sept. 14, 21, 28. [18] Lancet, Sept. 21. [19] N.Y. Med. J., Oct. 19. [20] Am. J. Med. Sc., Oct. [21] B. M. J., Nov. 2. [22] Lancet, Oct. 12. [23] Am. J. Med. Sc., Oct. [24] Med. Chron., Nov. [25] Int. Med. Mag., Oct. [26] Int. Med. Mag., Oct. [27] Am. J. Med. Sc., Nov. [28] Med. Rec., Oct. 19. [29] Ther. Gaz., Oct. 15. [30] Am. J. Med. Sc., Nov. [31] Lancet, Nov. 30. [32] Dublin J. Med. Sc., Nov. [33] Annals of Surg., Nov. [34] Am. J. M. Sc., Oct.

